# Predicting the protein-protein interactions using primary structures with predicted protein surface

**DOI:** 10.1186/1471-2105-11-S1-S3

**Published:** 2010-01-18

**Authors:** Darby Tien-Hao Chang, Yu-Tang Syu, Po-Chang Lin

**Affiliations:** 1 Department of Electrical Engineering, National Cheng Kung University, Tainan, 70101, Taiwan

## Abstract

**Background:**

Many biological functions involve various protein-protein interactions (PPIs). Elucidating such interactions is crucial for understanding general principles of cellular systems. Previous studies have shown the potential of predicting PPIs based on only sequence information. Compared to approaches that require other auxiliary information, these sequence-based approaches can be applied to a broader range of applications.

**Results:**

This study presents a novel sequence-based method based on the assumption that protein-protein interactions are more related to amino acids at the surface than those at the core. The present method considers surface information and maintains the advantage of relying on only sequence data by including an accessible surface area (ASA) predictor recently proposed by the authors. This study also reports the experiments conducted to evaluate a) the performance of PPI prediction achieved by including the predicted surface and b) the quality of the predicted surface in comparison with the surface obtained from structures. The experimental results show that surface information helps to predict interacting protein pairs. Furthermore, the prediction performance achieved by using the surface estimated with the ASA predictor is close to that using the surface obtained from protein structures.

**Conclusion:**

This work presents a sequence-based method that takes into account surface information for predicting PPIs. The proposed procedure of surface identification improves the prediction performance with an *F-measure *of 5.1%. The extracted surfaces are also valuable in other biomedical applications that require similar information.

## Background

The different types of interactions among proteins are essential to various biological functions in a living cell. Information about these interactions provides a basis to construct protein interaction networks and improves our understanding of the general principles of the functioning of biological systems [1]. Recent years have seen the development of various experimental techniques for systematic protein-protein interaction (PPI) analysis [2-5]. At present, however, experimentally detected interactions represent only a small fraction of the real interaction network [6,7]. Therefore, a number of computational approaches have been proposed to expedite the PPI detection process based on only experimental techniques [8].

Computational methods that depend on not only sequence information but also some prior knowledge of, for example, localization data [9], structural data [10,11], expression data [12,13] or information on the interactions of orthologs [14,15] cannot be applied on some essential proteins that are observed in most organisms [16]. To solve this problem, several sequence-based algorithms have been developed to detect potentially interacting protein pairs when no auxiliary information is available [17-23].

This work presents a novel sequence-based method which involves a mechanism for identifying the protein surface to help PPI prediction. This method employs the conjoint triad feature [24] for describing protein sequences and the relaxed variable kernel density estimator (RVKDE) [25] for classification. Conjoint triads, which treat three continuous amino acids as a single unit, have been shown to be a useful set of features in predicting protein-protein interactions [24]. This work improves this feature set by focusing on conjoint triads at the protein surface. This improvement is based on the assumption that protein-protein interactions are more related to amino acids at the surface than those at the core. To maintain the advantage of depending on only sequence information, this method employs an accurate accessible surface area (ASA) predictor, recently proposed by the authors [26], to determine the protein surface.

In this study, a collection of 691 PPIs is used to evaluate the prediction performance with and without the proposed mechanism for identifying the protein surface. The experimental results show that the surface information promotes PPI prediction based on feature encoding with conjoint triads. Furthermore, the quality of the predicted surface is analyzed using a number of protein structures collected from the Protein Data Bank (PDB) [27]. The experimental results demonstrate that the performance of PPI prediction achieved using the predicted surface is close to that achieved using the surface obtained from protein structures.

## Results and discussion

This section first describes the workflow of the proposed method. Next, the measurements and datasets for performance evaluation are presented. The proposed method is evaluated and compared with another sequence-based PPI predictor. At the end of the section, the predicted surface is compared to those obtained from protein structures.

### Proposed PPI prediction scheme

**Figure 1:**
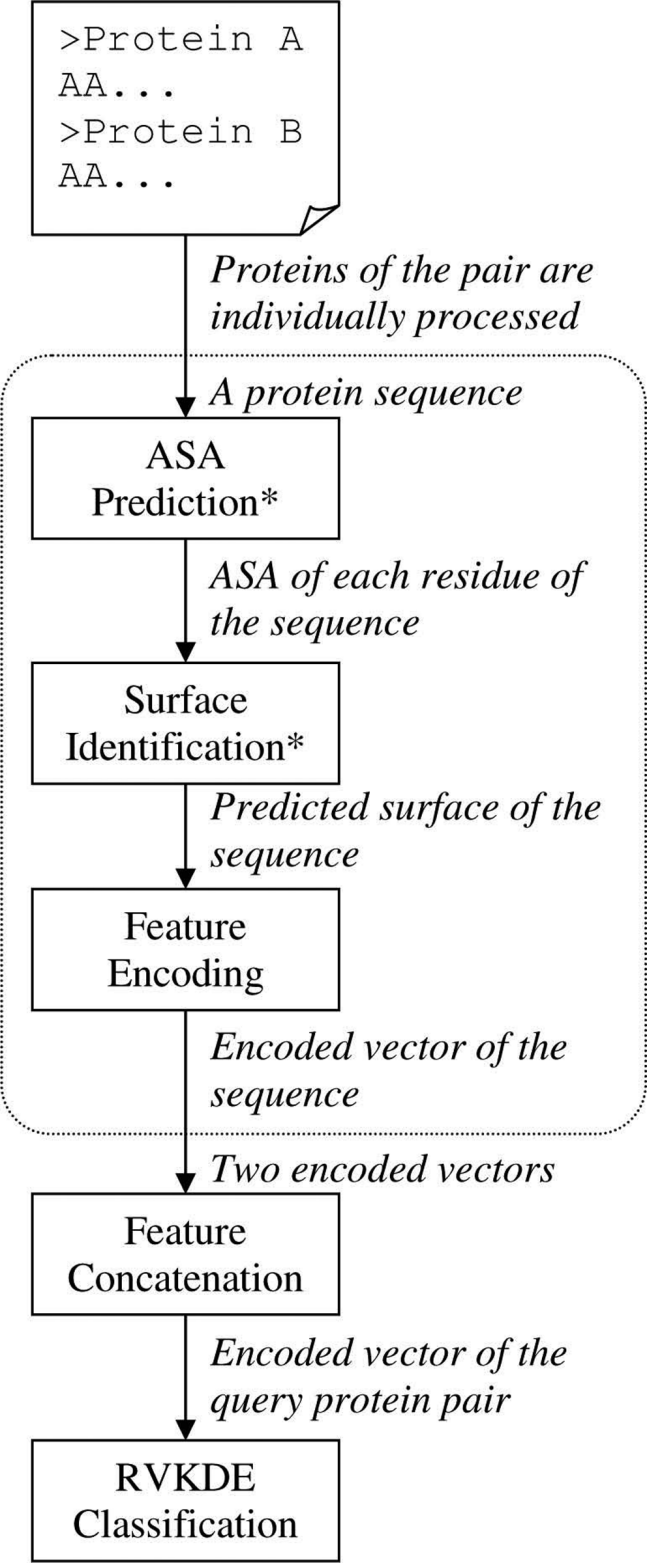
**Workflow of proposed method to predict interacting protein pairs**. Given a pair of protein sequences, this method first encodes each of the two sequences as a vector. The encoding process comprises three steps; the two steps marked with an asterisk are the major contributions of this work. The two vectors are concatenated as the feature vector of the protein pair and submitted to the RVKDE for classifying whether the two proteins have interactions.

Figure 1 depicts the workflow of the developed method. Steps marked with an asterisk indicate the major differences between the procedure in this work and those presented in previous PPI studies. First, the feature vectors of both proteins of a given protein pair are individually generated. This operation is further split into three steps: 'ASA Prediction', 'Surface Identification' and 'Feature Encoding'. The 'ASA Prediction' step invokes a sequence-based ASA predictor for assigning a relative ASA (RSA) value to each residue of the protein sequence. Based on these RSA values, the 'Surface Identification' step identifies surface sequence segments in which most residues have large RSA values. The detailed criterion of identifying surface segments is presented in the Methods section. Next, the 'Feature Encoding' step determines the frequencies of conjoint triads that are observed in the identified surface segments and uses these frequencies to generate the feature vector. Finally, the two feature vectors of the given protein pair are concatenated and sent to RVKDE for classifying whether the two proteins have interactions. See the Methods section for details of all of these steps.

### Measurements

Determining whether two proteins have interactions is a binary classification problem. Table 1 lists five measurements that are applied widely on evaluating binary classification problems. The *accuracy *is the most commonly used measurement, which represents an overall performance of a predictor. The *F-measure *is designed for problems where a class of instances attracts most attention, which is appropriate for PPI prediction [28]. The *precision *is the fraction of predicted interacting protein pairs that truly have interactions. The *sensitivity *is the fraction of interacting protein pairs correctly predicted to have interactions, while the *specificity *is the fraction of non-interacting protein pairs correctly predicted to have no interaction.

**Table 1:** Evaluation measurements.

Measurement	Abbreviation	Equation
*Accuracy*	*Acc*.	(TP+TN)/(TP+TN+FP+FN)
*F-measure*	*Fm*.	2TP/(2TP+FP+FN)
*Precision*	*Prec*.	TP/(TP+FP)
*Sensitivity *(*recall*)	*Sens*.	TP/(TP+FN)
*Specificity*	*Spec*.	TN/(TN+FP)

The definitions of the abbreviations used: TP is the number of interacting protein pairs correctly classified; FN is the number of interacting protein pairs incorrectly classified as non-interacting; TN is the number of non-interacting protein pairs correctly classified; and FN is the number of non-interacting protein pairs incorrectly classified as interacting.

### Datasets

A challenge in preparing protein-protein interaction datasets is the presence of some interactions that are observed in the laboratory experimentation but do not occur physiologically [6]. To ensure the quality of PPI data, an interaction should be consistent with other types of information [29], such as metabolomic [30] and gene-gene relationship data [31]. Though these types of data are often incomplete in most organisms at present, the interaction network of transcription factors (TF) of *Saccharomyces cerevisiae *is an extensively studied system in which all of such information are currently available [29]. Therefore, this study collects 691 interactions of 211 yeast TFs from several studies and databases [32-36] to generate a PPI dataset, SC691. In this dataset, the 691 interactions are used as positive instances, while other protein pairs created by coupling the 211 TFs are used as negative instances.

### Evaluation of PPI prediction

In the experiment, the SC691 dataset is randomly split into three subsets of 341, 175 and 175 interacting pairs. These subsets also contain 341, 175 and 175 non-interacting pairs obtained by arbitrarily sampling of the negative instances in the SC691 dataset. Care is taken to ensure that different subsets will not share identical instances. In this experiment, the first subset is used as the training set to predict the other two subsets. The predicted results of the second subset are used for parameter selection, while the predicted results of the third subset indicate the prediction performance of a PPI predictor. Therefore, an evaluation process is performed by first using the first subset to predict the second subset. Then the parameters that maximize the *F-measure *are used to predict the third subset. Since the procedure for generating these subsets involves randomness, the evaluation process is performed ten times to eliminate the evaluation bias in a single evaluation process.

Table 2 presents the prediction performance of the proposed method under various surface conditions. In this work, the predicted surface is union of several surface sequence segments of fixed length. The parameter *o *restricts the minimum number of surface residues in a surface segment, and thereby affects the predicted surface. See the 'Surface identification' subsection for details. Table 2 also includes the prediction performance of the sequence-based method proposed by Shen *et al*. [24], which uses conjoint triads that are observed in protein sequences without considering surface information. In Table 2, all the five measurements of are improved after introducing the surface information without depending on the surface condition. Considering surface segments that include at least three surface residues achieves the best performance, and the other three surface conditions deliver similar performance. This suggests that to form a stable interface requires at least three residues. Restricting that a surface segment must have at least four surface residues would be too rigorous and filter out some potential surface segments.

**Table 2:** Performance achieved by considering and by neglecting surface information.

	*Acc*. (%)	*Fm*. (%)	*Prec*. (%)	*Sens*. (%)	*Spec*. (%)
Without surface information					
Shen *et al*.'s work	68.2 ± 4.3	70.4 ± 3.2	66.4 ± 5.1	75.4 ± 5.4	61.0 ± 10.2
Surface identified using different *o*					
1	72.3 ± 1.4	73.7 ± 1.6	70.3 ± 2.3	77.8 ± 4.4	66.9 ± 5.1
2	72.1 ± 3.2	74.0 ± 2.2	69.7 ± 4.2	79.3 ± 3.7	64.9 ± 8.3
3	74.1 ± 2.0	75.5 ± 2.0	71.8 ± 2.4	79.7 ± 3.5	68.6 ± 4.0
4	71.7 ± 3.8	73.4 ± 2.3	69.8 ± 4.9	77.9 ± 5.9	65.4 ± 11.5

Parameter *o *restricts the minimum number of surface residues in a surface sequence segment.

As a result, the average *Acc*., *Fm*., *Prec*., *Sens*. and *Spec*. of the developed method are 74.1%, 75.5%, 71.8%, 79.7% and 68.6%, respectively. All five measurements are superior to those delivered by the predictor without surface information. These results show that the proposed mechanism for identifying the protein surface helps to predict protein-protein interactions based on feature encoding with conjoint triads.

### Evaluation of predicted surface

As shown in Figure 1, the 'ASA Prediction' and 'Surface Identification' steps are the major differences between this work and others. To evaluate the added components, this subsection reports the experiment for answering two questions: a) how the predicted surface overlap with the surface obtained from protein structures and b) how the PPI prediction performs when using the predicted surface compared to those using the surface obtained from protein structures. The ten TFs from the SC691 dataset that have structures in PDB (Table 3) are used to generate a smaller dataset. This dataset, called SC85, includes 85 positive and 1980 negative instances from the SC691 dataset. Each pair of the SC85 dataset contains at least one of the ten TFs. In this experiment, a prediction is made by five-fold cross validation of the SC85 dataset, in which each fold includes 17 positive and 396 negative instances. The cross validation is performed ten times to eliminate the evaluation bias. The surface condition is set to consider surface segments that include at least three surface residues.

**Table 3:** Proteins in the SC691 dataset that have structures in PDB

Name	Description	PDB ID: chain
SPT4	Transcription initiation protein	2EXU:A
GAL80	Galactose/lactose metabolism regulatory protein	3BTV:A
MED18	RNA polymerase II mediator complex subunit 18	2HZM:B
MED20	RNA polymerase II mediator complex subunit 20	2HZM:A
MED21	RNA polymerase II holoenzyme component SRB7	1YKE:B
MTF1	Mitochondrial replication protein	1I4W:A
NHP6A	Nonhistone protein 6A	1CG7:A
PHO80	Cyclin, negatively regulates phosphate metabolism	2PMI:B
TOA1	Transcription initiation factor IIA large chain	1RM1:C
TOA2	Transcription initiation factor IIA small chain	1RM1:B

Table 4 shows the overlap of the predicted surface and the surface obtained from protein structures, called 'structural surface', in the residue level. The predicted surface is identified based on the predicted ASA obtained from the adopted ASA predictor, while the structural surface is identified based on the actual ASA obtained by invoking the Dictionary of Protein Secondary Structure (DSSP) program [37]. In this experiment, at least 75% (91.9% in average) of surface residues--residues in the structural surface--are included in the predicted surface. Conversely, some individual trials delivered <60% *specificity*, and the average *specificity *(77.7%) is relative lower in comparison with the *sensitivity*. These results indicate that a certain percentage of buried residues--residues outside the structural surface--are incorrectly included in the predicted surface. Namely, the proposed method delivers a larger surface than that obtained based on actual ASA. Overall, the predicted surface is consistent to structural surface in this dataset according to the *accuracy *and *F-measure*.

**Table 4:** Overlap between predicted and structural surface.

	*Acc*. (%)	*Fm*. (%)	*Prec*. (%)	*Sens*. (%)	*Spec*. (%)
Trial 1	76.1	81.3	76.0	87.3	59.7
Trial 2	76.3	81.2	71.1	94.5	54.9
Trial 3	78.6	82.3	74.2	92.5	62.3
Trial 4	77.2	81.3	83.3	79.4	73.7
Trial 5	95.1	96.2	92.7	100.0	86.9
Trial 6	88.0	90.2	82.2	100.0	73.3
Trial 7	95.1	96.2	92.7	100.0	86.9
Trial 8	95.1	96.2	92.7	100.0	86.9
Trial 9	83.0	83.1	92.4	75.4	92.4
Trial 10	93.4	94.7	100.0	90.0	100.0
Overall	85.8 ± 8.4	88.3 ± 7.0	85.7 ± 9.7	91.9 ± 9.0	77.7 ± 15.2

The equation of each measurement is identical to Table 2, while the definitions of the abbreviations used are different: TP is the number of residues within the structural surface and are correctly included in the predicted surface; FN is the number of residues within the structural surface but are incorrectly excluded from the predicted surface; TN is the number of residues outside the structural surface and are correctly excluded from the predicted surface; and FN is the number of residues outside the structural surface but are incorrectly included in the predicted surface.

The next analysis aims to elaborate how much does the difference between predicted and structural surface affect the results of PPI prediction. Table 5 presents the performance of PPI prediction using the predicted and structural surface. Though the predicted surface performs worse than the structural surface, the differences in all evaluation measures are less than the standard deviations of using the structural surface. These results reveal that the added components of this work can achieve comparable performance of dealing yeast TFs to that delivered using structure information.

**Table 5:** Performance achieved using predicted and structural surface.

	*Acc*. (%)	*Fm*. (%)	*Prec*. (%)	*Sens*. (%)	*Spec*. (%)
Predicted surface					
Trial 1	96.2	38.1	58.5	28.2	99.1
Trial 2	96.2	40.9	57.4	31.8	99.0
Trial 3	96.1	38.2	54.3	29.4	98.9
Trial 4	96.3	38.7	61.5	28.2	99.2
Trial 5	96.6	40.3	70.6	28.2	99.5
Trial 6	96.4	43.1	62.2	32.9	99.1
Trial 7	96.7	41.0	75.0	28.2	99.6
Trial 8	96.4	40.9	61.9	30.6	99.2
Trial 9	94.7	37.5	36.3	38.8	97.1
Trial 10	95.7	39.7	47.5	34.1	98.4
Overall	96.1 ± 0.6	39.8 ± 1.7	58.5 ± 11.0	31.1 ± 3.5	98.9 ± 0.7
Structural surface					
Trial 1	96.0	39.7	52.9	31.8	98.8
Trial 2	96.6	41.3	69.4	29.4	99.4
Trial 3	96.1	40.3	55.1	31.8	98.9
Trial 4	96.3	39.7	61.0	29.4	99.2
Trial 5	96.3	40.3	59.1	30.6	99.1
Trial 6	96.4	42.7	60.9	32.9	99.1
Trial 7	96.2	41.5	56.0	32.9	98.9
Trial 8	96.5	42.5	64.3	31.8	99.2
Trial 9	95.9	38.8	50.0	31.8	98.6
Trial 10	96.0	40.3	51.9	32.9	98.7
Overall	96.2 ± 0.2	40.7 ± 1.3	58.1 ± 6.0	31.5 ± 1.3	99.0 ± 0.3

ASA values of predicted surface are obtained from the adopted ASA predictor. ASA values of structural surface are obtained using the DSSP program.

**Figure 2:**
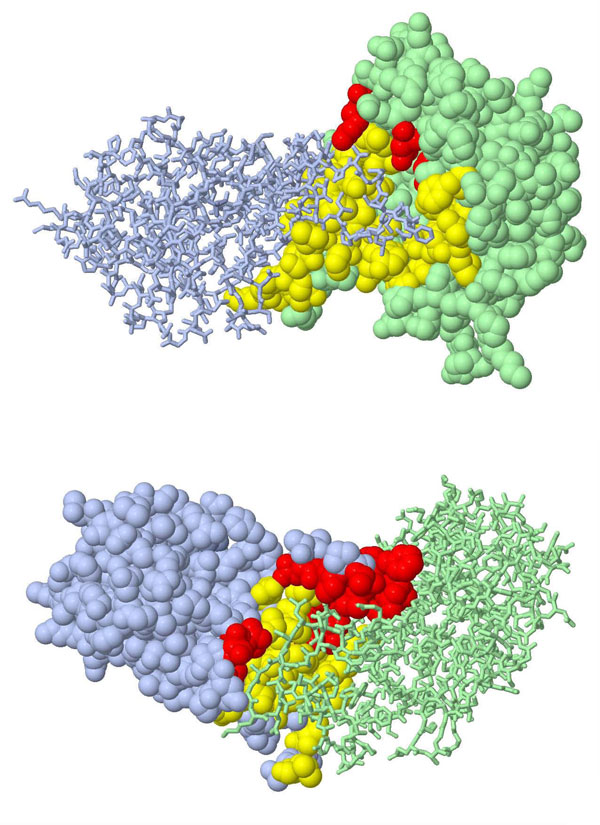
**Example of the surface predicted by the present method**. This example employs the two subunits of RNA ploymerase II (PDB ID: 2HZM), Med18 (chain B) and Med20 (chain A), to show the predicted surface relative to the interface residues. The protein chain in *spacefill *mode is the target subunit used in surface identification; the protein chain that is displayed in *stick *mode is treated as the interacting partner of the target subunit. The predicted surface that overlaps the interface residues is shown in yellow, and the non-overlapping region is shown in red. Med18 is the target subunit in (a), and Med20 is the target subunit in (b).

In the end of this section, a protein pair from the collected 691 PPIs of which both the proteins appear in the same complex structure in PDB is used to plot the overlap between the predicted surface and the interface. This complex (PDB ID: 2HZM) includes the two subunits (Med18 and Med20) of the RNA ploymerase II, which is central to eukaryotic gene expression and has been studied extensively [38]. Figure 2 presents the interface residues of Med18 (chain B in 2HZM) and Med20 (chain A in 2HZM). Interface residues are defined as those that have at least one heavy atom within 5 Å distance of the interacting partner. This definition is similar to those used in many studies [39-41].

For Med18, the present method successfully excludes 80 (accounting for ~26.1%) from total 307 residues while preserving 48 (accounting for ~92.3% of the 52) interface residues. As shown in Figure 2(a), most interface residues, specified in yellow, are included. However, for Med20, the proposed method misses 24 (accounting for ~54.5% of the 44) interface residues in the predicted surface in Figure 2(b). Figure 2(b) reveals that the predicted surface misses the segment (residues 86-107) of Med20 that acts like an arm stretching to Med18. A comparison with the interface shown in Figure 2(a) suggests that the present method may perform better at handling flatter interfaces. Since protein subunits may interact and form relatively flat or twisted surfaces [42], the good performance of the present method probably results from the fact that most of the collected *S. cerevisiae *TFs have relatively flat surfaces.

These results also reveal that the proposed mechanism for identifying the surfaces of proteins with relatively twisted surfaces must be improved.

## Conclusion

An enormous gap exists between the number of protein structures and the huge number of protein sequences. Hence, predicting protein functions directly from amino acid sequences remains one of the most important problems in life science. This work presents a computational approach for PPI prediction based on only sequence information. Notably, a mechanism of extracting surface information is proposed to refine the feature vector for representing a protein sequence. This method is analyzed in terms of a) the performance in predicting PPIs and b) the quality of the predicted surface. The experimental results show that the present method improves on the prediction performance of PPI with an *F-measure *of 5.1%. Furthermore, the predicted surface of yeast TFs is consistent with that obtained from structures, which encourages applying the present steps of surface identification in other biomedical problems that require similar information.

## Methods

### ASA prediction

**Figure 3:**
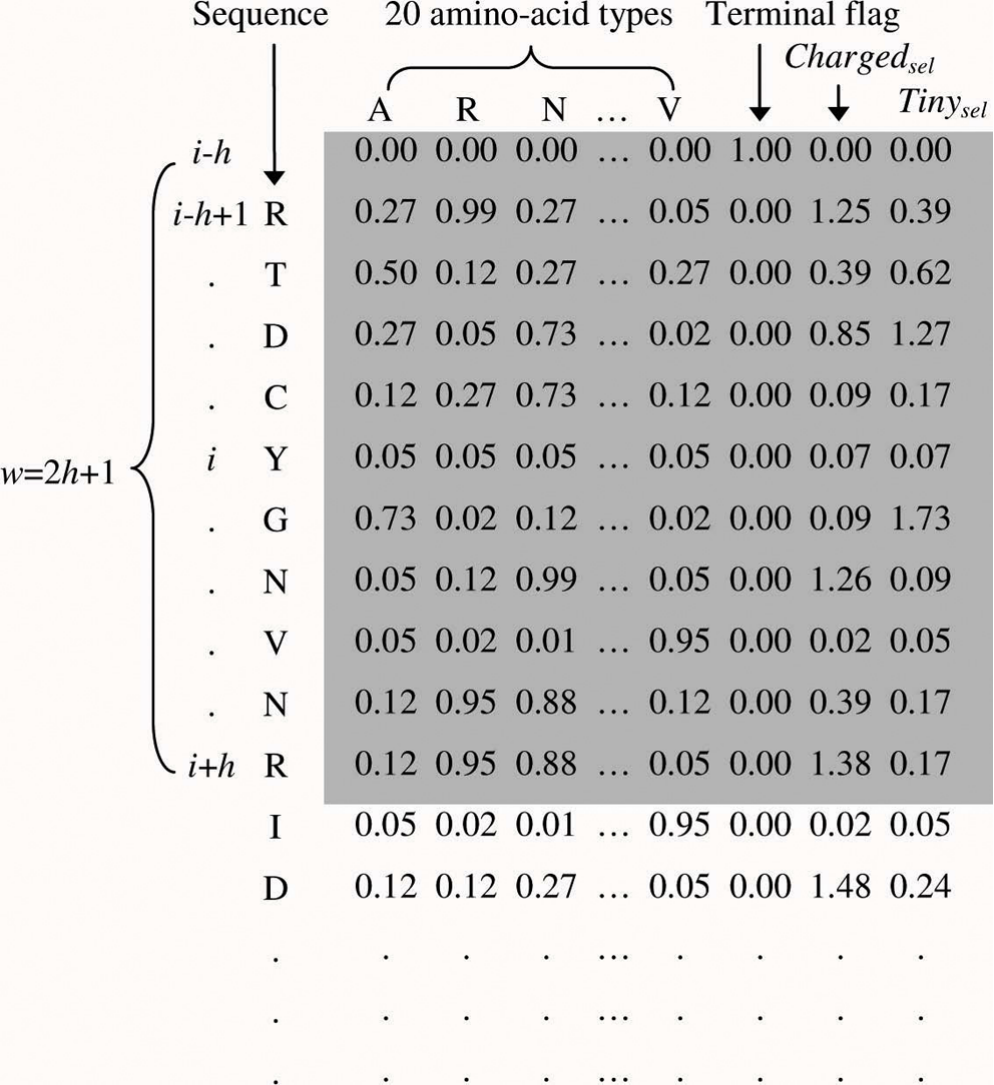
**Example of encoding a residue in the PSSM-2SP form**. This example encodes the fifth residue (*i *= 5) of a protein (PDB ID: 154L) with window size 11 (*w *= 11 and *h *= 5). A position is represented by a 23-dimensional vector (20 amino acid values, a terminal flag and two group values). The first row is a pseudo terminal residue where only the terminal flag is 1 and all 22 other values are zero. Finally, the *i*-th residue is encoded with its neighboring positions to form a 253-dimensional feature vector.

This study adopts two cascading regressions to predict relative ASA (RSA) values. The first stage uses the PSSM-2SP (stands for position specific scoring matrix with two sub-properties) profile [26] to encode a protein sequence. The PSSM-2SP profile is an enhanced PSSM profile, which describes the likelihood of a particular residue substitution at a specific position based on evolutionary information [21]. The construction of the PSSM profile is achieved by first invoking the PSI-BLAST program [43] to the non-redundant (NR) database obtained from the NCBI. The PSSM-2SP profile adds more two accumulated profile values according to residue groups *Charged*_*sel *_(K and D) and *Tiny*_*sel *_(A and G). The resulting PSSM-2SP profile is rescaled to [0,1], using the following logistic function [44]:

where *x *is the raw value in the PSSM profile and *x' *is the value corresponding to *x *after rescaling. Finally, we add a terminal flag and format the profile into the vector representation with a window size *w*_1 _(*w*_1 _= 11 in our implementation). Figure 3 shows an example of encoding a residue to its corresponding PSSM-2SP form.

The second stage encodes a protein sequence based on neighboring solvent accessibility [26,45]. The *i*-th residue in a protein sequence is represented as a 2*w*_2_+1 dimensional vector **v **= (*a*_*i*-*h*_, *t*_*i*-*h*_, *a*_*i*-*h*__+*1*_, *t*_*i*-*h*+*1*_, ..., *a*_*i*_, *t*_*i*_, ..., *a*_*i*+*h*_, *t*_*i*+*h*_, *l*), where *a*_*i *_is the predicted RSA value of the *i*-th residue in the first regression, *t*_*i *_is the terminal flag as either 1 (a null/terminal residue) or 0 (otherwise), *l *is the sequence length and *w*_2 _= 2*h*+1 is window size (*w*_2 _= 5 in our implementation).

The support vector regression (SVR) is used as the regression tool for both stages. The SVR is a kernel regression technique that constructs a model based on support vectors. This model expresses *y *as a function of **v **with several parameters:

where *K*() is the kernel function, and *b *and *w*_*i *_are numerical parameters determined by minimizing the prediction error on training samples. The problem is to find the support vectors and determine parameters *b *and *w*_*i*_, which can be solved by constrained quadratic optimization [46]. The LIBSVM package (version 2.86) [47] is used for SVR implementation in this study.

### Surface identification

The employed ASA predictor makes predictions at the residue level. The predicted RSA value of each residue enables surface residues to be defined as those whose RSA values are equal to or larger than a threshold *t*. These identified surface residues are frequently scattered throughout the protein sequences. This work develops a process for generating a set of surface segments each of which is a consecutive sub-sequence of minimum length. Because a conjoint triad represents three continuous amino acids, these consecutive segments are more suitable than scattered surface residues for being encoded with conjoint triads.

**Figure 4:**
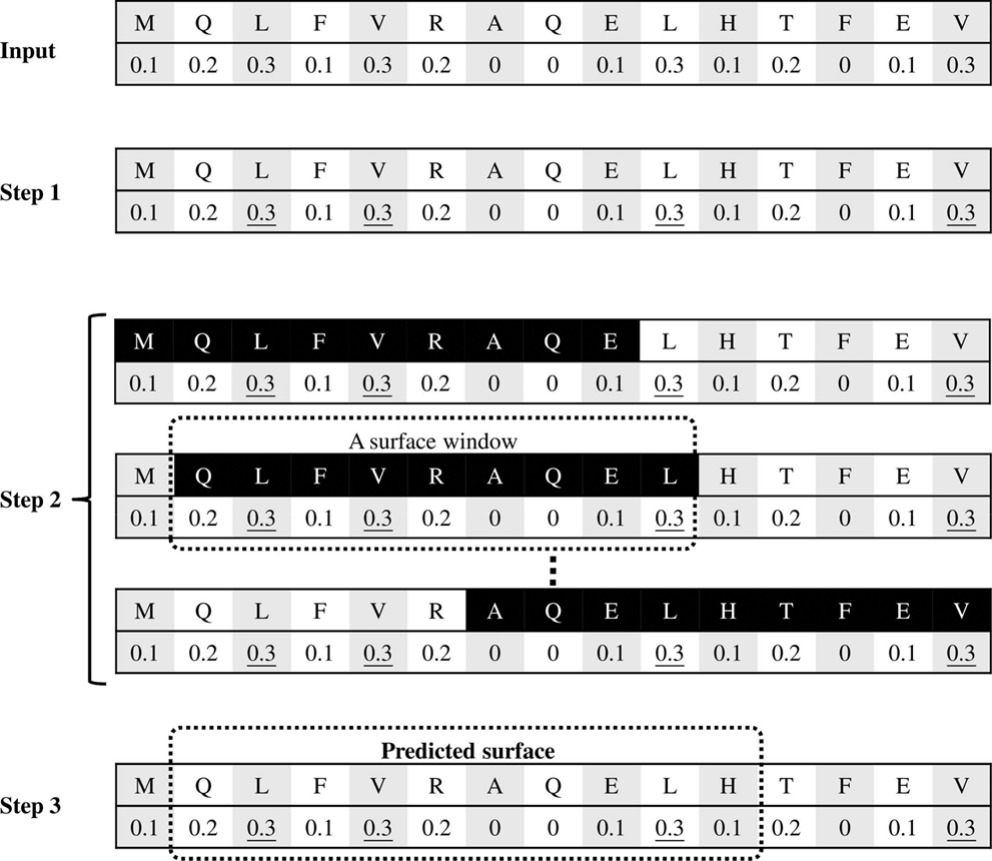
**Identifying surface of protein sequence**. Input: Each residue of the sequence is associated with a predicted RSA value. Step 1: Identify surface residues having RSA values ≥*t*. Step 2: Scan the sequence with a sliding window of size *w*, where each surface window must include at least *o *surface residues. Step 3: Predicted surface is union of all surface windows. *t *= 0.3, *w *= 9 and *o *= 3 in this example.

Figure 4 depicts the process of surface identification. The present method uses a sliding window of size *w *to scan the protein sequence. A sliding window is identified as a surface window if it contains at least *o *surface residues. Finally, the predicted surface is the union of all surface windows. In this study, *t *and *w *are parameters to be set either by cross-validation or by the user, while *o *is suggested to be three according to the experiment results.

### Feature encoding

Based on the design by Shen *et al*. [24], this work encodes each protein sequence as a feature vector by considering the frequencies of conjoint triads of that protein sequence. An amino acid triad regards is a unit of three continuous amino acids. Each PPI pair is thus encoded by concatenating the two feature vectors of the two individual proteins of that pair. The 20 amino acids are clustered into seven groups (Table 6) based on their dipoles and side chain volumes.

**Table 6:** Amino acid groups used herein.

**Group no**.	Amino acids
1	Ala, Gly, Val
2	Ile, Leu, Phe, Pro
3	Tyr, Met, Thr, Ser
4	His, Asn, Gln, Tpr
5	Arg, Lys
6	Asp, Glu
7	Cys

**Figure 5:**
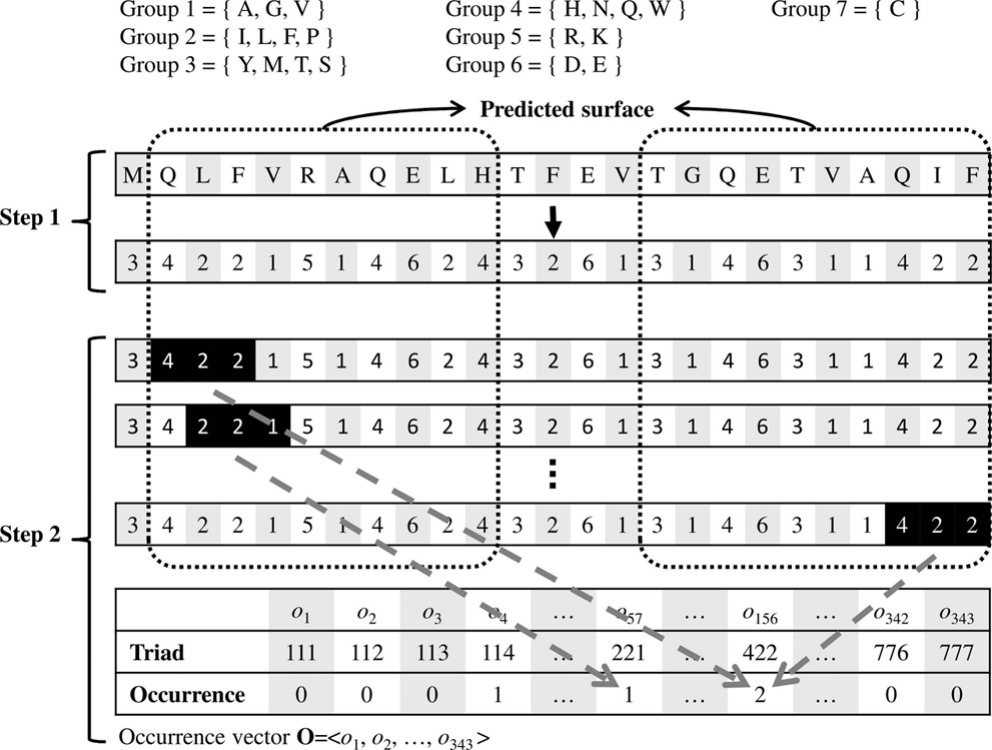
**Encoding a protein sequence as a feature vector using conjoint triads**. Step 1: Transform the amino acid sequence into the group sequence. Step 2: Scan the predicted surface along the group sequence, and count the triads in the occurrence vector **O**.

Figure 5 depicts the process of encoding a protein sequence. First, the protein sequence is transformed into a group sequence. This method then scans the predicted surface along the group sequence. Each scanned triad is counted in an occurrence vector, **O**, of which each element *o*_*i *_represents the number of the *i*-th type of triad observed in the predicted surface. The major contribution of this work is to ignore the occurrences of conjoint triads outside the predicted surface. The two vectors of both sequences of a pair of proteins are concatenated to form a 686-dimensional feature vector.

### Relaxed variable kernel density estimator

The relaxed variable kernel density estimator (RVKDE) [25] is used as the classification tool for PPI prediction. A kernel density estimator is in fact an approximate probability density function. Let {**s**_1_, **s**_2_... **s**_*n*_} be a set of sampling instances randomly and independently taken from the distribution governed by *f*_*X *_in the *m*-dimensional vector space. Then, with the RVKDE algorithm, the value of *f*_*X *_at point **v **is estimated as follows:

1) ;

2) *R*(**s**_*i*_) is the maximum distance between **s**_i _and its *ks *nearest training instances;

3) Γ(·) is the Gamma function [48];

4) ***β ***and *ks *are parameters to be set either through cross-validation or by the user.

When using RVKDE to predict protein-protein interactions, two kernel density estimators are constructed to approximate the distribution of interacting and non-interacting protein pairs, respectively. A query protein pair (represented as the feature vector **v**) is predicted to the class that gives the maximum value among the two likelihood functions defined as follows:

where |*S*_*j*_| is the number of class-*j *training instances, and (**v**) is the kernel density estimator corresponding to class-*j *training instances. In this study, *j *is either 'interacting' or 'non-interacting'. Current RVKDE implementation includes only a limited number, denoted by *kt*, of the nearest class-*j *training instances of **v **while computing (**v**) in order to improve the efficiency of the predictor. The *kt *is also a parameter to be set either through cross-validation or by the user.

## Competing interests

The authors declare that they have no competing interests.

## Authors' contributions

Author DTHC designed the methodology and conceived of this study. YTS and BCL designed the experiments and performed all calculations and analyses. All authors have read and approved this manuscript.
